# Photosynthetic Efficiency of *Marchantia polymorpha* L. in Response to Copper, Iron, and Zinc

**DOI:** 10.3390/plants12152776

**Published:** 2023-07-26

**Authors:** Carlo Sorce, Erika Bellini, Florinda Bacchi, Luigi Sanità di Toppi

**Affiliations:** 1 Department of Biology, University of Pisa, via L. Ghini, 13, 56126 Pisa, Italy; carlo.sorce@unipi.it (C.S.);; 2 Department of Biology and Biotechnology “Charles Darwin”, Sapienza University of Rome, Piazzale A. Moro, 5, 00185 Rome, Italy

**Keywords:** chlorophyll fluorescence, copper, iron, JIP test, *Marchantia polymorpha*, zinc

## Abstract

Metal micronutrients are essential for plant nutrition, but their toxicity threshold is low. In-depth studies on the response of light-dependent reactions of photosynthesis to metal micronutrients are needed, and the analysis of chlorophyll *a* fluorescence transients is a suitable technique. The liverwort *Marchantia polymorpha* L., a model organism also used in biomonitoring, allowed us to accurately study the effects of metal micronutrients in vivo, particularly the early responses. Gametophytes were treated with copper (Cu), iron (Fe) or zinc (Zn) for up to 120 h. Copper showed the strongest effects, negatively affecting almost the entire light phase of photosynthesis. Iron was detrimental to the flux of energy around photosystem II (PSII), while the acceptor side of PSI was unaltered. The impact of Fe was milder than that of Cu and in both cases the structures of the photosynthetic apparatus that resisted the treatments were still able to operate efficiently. The susceptibility of *M. polymorpha* to Zn was low: although the metal affected a large part of the electron transport chain, its effects were modest and short-lived. Our results may provide a contribution towards achieving a more comprehensive understanding of response mechanisms to metals and their evolution in plants, and may be useful for supporting the development of biomonitoring techniques.

## 1. Introduction

Heavy metal pollution is a growing concern for both human and ecosystems’ health. Natural processes release these elements from the Earth’s crust, but anthropogenic activities are responsible for dispersing large amounts of them worldwide [1,2]. The distinctive feature of heavy metals is their density, which exceeds 5 g cm^−3^ [3]. Beyond being very persistent in the environment, most of them are toxic for all living organisms [4,5]. Some heavy metals are instead essential for plant nutrition if acquired in small levels, otherwise they can easily become harmful when absorbed in amounts exceeding the plant’s physiological needs [6]: in this case, they may also induce the overproduction of reactive oxygen species (ROS), which target key biological molecules [2]. Further negative effects of heavy metals are osmotic stress, plasmolysis, extreme vacuolation of cells and accumulation of excess starch [4,7,8,9].

Several heavy metals also adversely affect photosynthesis, in diverse ways. These elements may lower the number and size of chloroplasts and disrupt thylakoid arrangement [10,11,12,13]. Stomatal and mesophyll conductance for CO_2_ decline in the leaves of plants growing in heavy metal-contaminated soils [14,15]. The concentration of photosynthetic pigments decreases following exposure to heavy metals, owing to chlorophyll biosynthesis repression; moreover, chlorophyll function may be impaired by the substitution of Mg in the center of the porphyrin ring [16,17,18]. The whole photosynthetic electron transport chain can be severely affected by heavy metals [15] and photosystem II (PSII) is particularly vulnerable. Both the donor and the acceptor side of PSII may be damaged by excessive concentrations of some metal nutrients like, for example, Cu and Zn [19,20,21,22].

Knowledge of the functioning of light-dependent reactions of photosynthesis can be acquired by measuring chlorophyll a fluorescence (ChlF). The analysis of the fluorescence transients is a reliable technique based on high-rate recordings made on dark-adapted photosynthetic organs during one second of exposure to a saturating red light. Fifty µs after the start of illumination the fluorescence value is designated “F_O_” and corresponds to the status of all reaction centers (RCs) of PSII open, when the primary quinone acceptor Q_A_ is fully oxidized. At the end of the illumination period ChlF reaches the peak value F_P_, which coincides with F_M_ if light is saturating, i.e., if it induces the closure of all RCs. When represented on a logarithmic time scale, the fluorescence transient from F_O_ to F_P_ shows a polyphasic pattern, known as the OJIP curve. The acronym refers to the O, J, I, P steps at 50 μs, 2 ms, 30 ms and the peak value of ChlF. The rise of ChlF emission is attributed to the progressive reduction of Q_A_ (i.e., to RC closure). During the O–J phase, Q_A_ are reduced only once and they begin to be re-oxidized at the state J, when electron transfer to Q_B_ starts. The J–I phase is determined by the balance between reduction and oxidation of the PQ pool and the I–P phase is related to electron transfer through PSI up to the final transporters on the acceptor side of PSI. The physiological status of the plant strongly influences the shape of the OJIP curve, but more precise information can be gained by using the JIP test, which models the light-driven bioenergetic reactions of photosynthesis through the elaboration of ChlF values recorded at specific steps, particularly at 50 µs (F_O_ or state O), 100 µs (F_100_), 300 µs (F_300_), 2 ms (state J), 30 ms (state I) and at peak emission (F_M_, state P). The elaboration yields biophysical parameters that quantify the functioning of PSII (with higher level of detail) and of the rest of the photosynthetic electron transport chain. The JIP-test is based on the theory of energy fluxes in biomembranes [23]. The measured ChlF values and the calculated JIP test parameters that are discussed in the present work are listed and defined in Table 1. The energy flux starts with the absorption of light by PSII antenna complexes (ABS), followed by the reduction of the primary electron acceptor Q_A_ (“trapping”, TR), the electron transport further than Q_A_^−^ (“electron transport”, ET) and finally the reduction of the end electron transporters on the acceptor side of PSI (RE). At each step part of the energy is dissipated; therefore, the efficiency of energy conservation can be expressed as quantum yields φ (efficiencies on the basis of light absorption; i.e., fluxes per ABS), as efficiencies per TR (ψ) and as efficiency per ET (δ). Expressions of these independent steps contributing to photosynthesis have been calculated, and also combined to yield both single- and overall performance indexes [24,25]. Energy fluxes are also calculated and expressed as specific energy fluxes per active PSII RC and as phenomenological energy fluxes per excited cross section of PSII. This technique provides detailed information on the structure and function of the photosynthetic apparatus, especially of PSII [26].

The response to metal micronutrients is an actively investigated subject, also because it contributes to represent the theoretical foundation of biomonitoring and phytoremediation. The effects of heavy metal stress on photosynthesis have been studied using both in vitro and in vivo systems. The latter grants more reliable evidence, provided that the administered metals are efficiently absorbed and translocated into the chloroplast. However, in higher plants, when heavy metals reach inhibitory concentrations in leaves, roots may have already undergone severe toxic effects, therefore the observed alterations of the photosynthetic process might be only an indirect consequence of the exposure to a metal. Investigations carried out on bryophytes are not affected by this drawback, because these plants can rapidly absorb large amounts of heavy metals through their whole gametophyte, owing to their high surface/volume ratio, elevated cation exchange capacity and lack of strong hydrophobic barriers: in this way, the response of photosynthesis to heavy metals can be accurately studied in vivo, even shortly after the beginning of treatments. Because of their morphofunctional features and their worldwide distribution, bryophytes are employed for heavy metal pollution biomonitoring, but there is still the need to deepen our knowledge on this topic. Indeed, the impact of heavy metals, and particularly of high concentrations of metal micronutrients, on the physiology of bryophytes has been poorly characterized [27]. Among them, the thalloid liverwort *Marchantia polymorpha* L. (Marchantiophyta) represents a crucial step in plant phylogeny [28,29] and is a valuable model organism in functional, molecular, and evolutionary studies on land plants, as well as an excellent tool for biomonitoring [30,31]. Nevertheless, there is limited knowledge about the responses of *M. polymorpha* to regulate and cope with high concentrations of metal micronutrients. In recent years, an article has shed light on the effects of elevated toxicity in *Marchantia polymorpha* when exposed to high concentrations of Cu and Zn (0.2 and 2 mM) over a long exposure time [31]. To help bridge this knowledge gap, our work is aimed at investigating in depth the response of *M. polymorpha* photosynthesis light reactions to excessive amounts of some metal micronutrients, namely Cu, Zn and Fe. Experimental evidence on this topic is scant, but it is crucial to achieve a thorough understanding of the mechanisms involved in metal detoxification, for the development of effective biomonitoring techniques that rely on the application of this species in environmental studies with heavy metals. Moreover, knowledge of physiological responses of photosynthesis to metal micronutrients may pave the way to biochemical and molecular studies on the evolution of metal tolerance in land plants. The outcome of exposure to heavy metals may be effectively investigated by analyzing ChlF, which is particularly suitable for revealing stress responses right from the early stages.

## 2. Results

The effects of treatments with excess heavy metals have been quantified using the parameters described in Table 1, most of which are the results of JIP test. The effects of each metal at each concentration and for each treatment time were compared with the respective controls. Below are described only the results related to the metal-concentration-time combinations that had significant effects. Data interpretation is based mostly on [32,33,34,35].

### 2.1. Responses to Excess Cu

**Figure 1:**
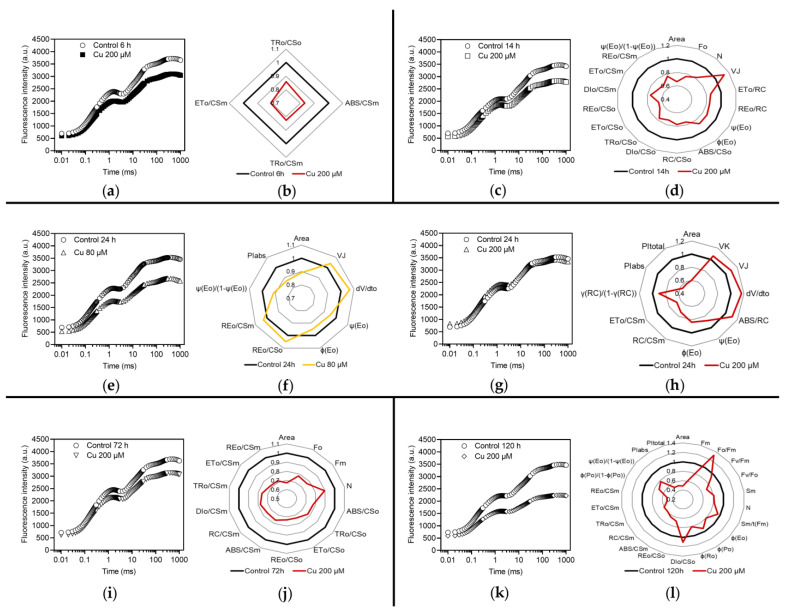
Effects of the exposure to 200 µM Cu for 6 (**a**,**b**), 14 (**c**,**d**), 24 (**g**,**h**), 72 (**i**,**j**) and 120 h (**k**,**l**), and to 80 µM Cu for 24 h (**e**,**f**) in dark-adapted *M. polymorpha* gametophytes. Induction transients of ChlF (**a**,**c**,**e**,**g**,**i**,**k**) and spider plots (**b**,**d**,**f**,**h**,**j**,**l**) of parameters of JIP test (described in Table 1), normalized to the values of the control, which were set as one. Black lines = control; red lines = 200 µM Cu; orange line = 80 µM Cu. Only those parameters that differed significantly from the control (*p* < 0.05) are shown. All values are the mean of nine replications.

Exposure of *M. polymorpha* for 6 h to Cu induced a significant response only at 200 µM concentration. Raw ChlF data followed a different pattern in treated gametophytes (Figure 1a) and significant differences were found between treated samples and the control in four parameters belonging to the group of phenomenological energy fluxes per excited cross section of active PSII: TR_0_/CS_O_, ABS/CS_M_, ET_0_/CS_M_ and TR_0_/CS_M_ (Figure 1b).

The subscript letter following “CS” means that the parameter refers to the state of all RCs open (subscript “O”) or to the state of all RCs closed (subscript “M”). The treatment 200 µM Cu (Cu200) resulted in a decrease in energy absorption by the PSII antenna (ABS/CS_M_) and in energy transfer from Q_A_^−^ to the intersystem electron acceptors (ET_0_/CS_M_) when all RCs were closed. Furthermore, there was a lower energy trapping by active PSII units (TR_0_/CS_O_ and TR_0_/CS_M_), i.e., the reduction of Q_A_ was lessened (both when all RCs were open and when they were all closed).

The treatment Cu200 induced changes in the pattern of fluorescence transients after 14 h (Figure 1c) and broad negative effects were revealed by JIP test (Figure 1d), which demonstrated the alteration of many parameters belonging to all the main groups illustrated in Table 1.

The lower values of ABS/CS_O_ (≈F_O_) suggest a diminished absorption of photon energy per excited PSII cross section with open RCs, leading to weaker fluxes of energy dissipation (DI_0_/CS_O_, but it was evident also when RCs were closed: see the value of DI_0_/CS_M_) and trapping (TR_0_/CS_O_). There was a decrease in the number of active RCs (RC/CS_O_); consequently, their turnover number (N, i.e., the rate of their reduction and re-oxidation) declined. The contraction of the number of active RCs is substantiated by the lower value of Area, which is a proxy of the number of Q_A_ acceptors, and the higher value of V_J_, that provides an estimation of the number of closed RCs, i.e., of reduced Q_A_ molecules. The energy flux from Q_A_^−^ to the intersystem electron acceptors was severely affected in treated gametophytes, as demonstrated by many parameters (ET_0_/RC, ET_0_/CS_O_ and ET_0_/CS_M_). Furthermore, the efficiency with which a PSII trapped electron was transferred from Q_A_^−^ to the secondary quinone acceptor (Q_B_ or, particularly when it is not bound to PSII, plastoquinone, PQ) (ψE_0_), as well as the quantum yield of such transfer (φE_0_), were lessened by the treatment. The value of the performance index ψ(E_0_)/(1 − ψ(E_0_)) shows that the contribution of intersystem electron transport to the global performance of photosynthesis light reactions was negatively affected by the exposure to 200 µM Cu. Adverse effects were detected also on the transfer of energy from Q_A_^−^ to the end acceptors of PSI, both per active PSII (RE_0_/RC) and per excited PSII cross section (RE_0_/CS_O_ and RE_0_/CS_M_, respectively).

Exposure of gametophytes for 24 h to 80 µM Cu (Cu80) yielded significant effects, as suggested by the pattern of ChlF (Figure 1e) and JIP test (Figure 1f). The treatment Cu80 diminished the number of RCs, as shown by the decrease of Area, that led to a fast accumulation of reduced Q_A_ (higher V_J_ and (ΔV/Δt)_0_). Treated gametophytes partially lost their efficiency of transferring the trapped energy from Q_A_^−^ to Q_B_ (decreased ψE_0_ and φE_0_), which conceivably lowered the performance of intersystem electron transport (ψ(E_0_)/(1 − ψ(E_0_))). The decrease of the performance index of energy conservation of absorbed photons up to Q_B_ reduction (PI_ABS_) confirmed that this treatment impacted mainly PSII and the energy flux around it, while the flux through the final part of the photosynthetic electron transport chain was increased (greater values of RE_0_/CS_O_ and RE_0_/CS_M_).

After 24 h, Cu200 also altered substantially the light reactions of photosynthesis. Fluorescence transients were not notably upset (Figure 1g), while JIP test (Figure 1h) highlighted several consequences of the treatment. Damage to the oxygen-evolving complex of PSII (OEC) was evident from the high value of V_K_. Impairments in RCs were demonstrated by the low Area, and high V_J_ plus (ΔV/Δt)_0,_ which means fewer total RCs and a greater number of reduced RCs. Energy flux in the intersystem declined (lower ET_0_/CS_M_) and became less efficient (decreased ψE_0_ and φE_0_). These alterations had a negative impact on the two performance indexes of energy conservation of absorbed photons (PI_ABS_ and PI_tot_), pointing at a general decline of the photosynthetic process. The rise of ABS/RC seems to reveal an increase in the apparent antenna size of active PSII, but actually the real cause is the decrease in the number of active RCs, as evidenced by the lower value of RC/CS_M_ and of γRC/(1 − γRC), which is the number of active RCs per antenna chlorophyll of PSII.

The gametophytes treated with 200 µM Cu for 72 h exhibited differences in the pattern of ChlF (Figure 1i) and JIP test (Figure 1j) provided many details about the gametophyte response to this treatment. Compared to the control, the lower values of ABS/CS_O_ (≈F_O_) and ABS/CS_M_ (≈F_M_) demonstrated a decreased absorption of photon energy per excited PSII cross section, likely causing weaker trapped exciton fluxes (TR_0_/CS_O_ and TR_0_/CS_M_), electron fluxes from Q_A_^−^ to Q_B_ (ET_0_/CS_O_ and ET_0_/CS_M_) and from the intersystem to the end acceptors of PSI (RE_0_/CS_O_ and RE_0_/CS_M_) per excited PSII cross section. With all RCs open, it was detected also a diminished flow of dissipated energy per excited cross section of PSII (DI_0_/CS_O_). The number of active RCs declined, as demonstrated by the lower Area; such decline was corroborated by the decreased number of active RCs per excited cross section of PSII (RC/CS_M_). The fewer active RCs were plausibly the reason for their diminished rate of reduction and re-oxidation (N).

The impact of 200 µM Cu was evident also after 120 h, as shown by the pattern of ChlF emission (Figure 1k) and by many parameters of JIP test (Figure 1l). The decrease in the F_V_/F_O_ value suggests that OEC was damaged, and the diminished values of F_M_ and ABS/CS_M_ indicate a lower photon flux absorbed by antenna complexes. Treated gametophytes lost part of their RCs (lower Area), a smaller part of these remained active (lower RC/CS_M_) and underwent slower turnover (lower N), with also a decreased average fraction of RCs that remained open in the time span from 0 to t(F_M_) (lower S_M_/t(F_M_)). In general, the whole electron transport capacity appeared downsized, with a reduced number of transporters per transport chain (S_M_). The quantum yield of primary photochemistry of PSII was reduced (F_V_/F_M_ and φP_0_), owing partly to the greater flow of dissipated energy per excited cross section of PSII (DI_0_/CS_O_), a process whose quantum yield (F_O_/F_M_) had also increased. The quantum yields of electron transport from Q_A_^−^ to Q_B_ (φE_0_) and of the overall electron transport up to the final acceptors of PSI (φR_0_) declined. The overall flux of energy per excited cross section of PSII was lessened: beyond the lower absorption of photons, reduced fluxes of trapped excitons (TR_0_/CS_M_), of electrons transferred from Q_A_^−^ to Q_B_ (ET_0_/CS_M_) and to the acceptor side of PSI (RE_0_/CS_O_ and RE_0_/CS_M_) were detected. The contributions of primary photochemistry reactions (φ(P_0_)/(1 − φ(P_0_))) and of intersystem electron transport (ψ(E_0_)/(1 − ψ(E_0_))) to the global performance of photosynthesis light reactions were diminished by the Cu treatment, with negative consequences on the performance indexes of energy conservation of absorbed photons (PI_ABS_ and PI_tot_).

### 2.2. Responses to Excess Fe

**Figure 2:**
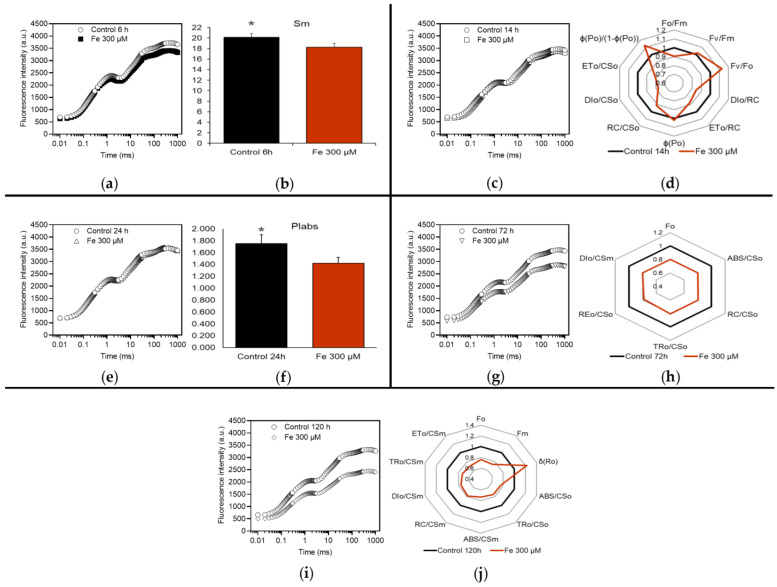
Effects of the exposure to 300 µM Fe for 6 (**a**,**b**), 14 (**c**,**d**), 24 (**e**,**f**), 72 (**g**,**h**) and 120 h (**i**,**j**), in dark-adapted *M. polymorpha* gametophytes. Induction transients of ChlF (**a**,**c**,**e**,**g**,**i**) and spider plots (**d**,**h**,**j**) or bar charts (**b**,**f**) of parameters of JIP test (described in Table 1); values in spider plots were normalized to those of the control, which were set as one. Black lines (or bars) = control; red lines (or bars) = 300 µM Fe. Only those parameters that differed significantly from the control (*, *p* < 0.05) are shown. All values are the mean of nine replications.

Exposure of *M. polymorpha* for 6 h to Fe induced a significant response only at 300 µM concentration (Fe300). Compared to the control, the ChlF emission differed only slightly and JIP test revealed only one significant difference between Fe300 and the control (Figure 2a,b, respectively): in the treated gametophytes S_M_ was lower, indicating a decrease of the number of electron carriers per electron transport chain.

Also, at 14 h the sole effective treatment was Fe300. Raw ChlF data of control and treated samples were similar (Figure 2c), while JIP test (Figure 2d) revealed several differences. A greater activity of OEC was demonstrated by the higher value of F_V_/F_O_, whereas the flux of electrons transferred from Q_A_^−^ to Q_B_ showed a decline, both when expressed per active PSII (ET_0_/RC) and per excited cross section of PSII (ET_0_/CS_O_). The dissipation of the absorbed energy decreased (lower F_O_/F_M_, DI_0_/RC and DI_0_/CS_O_). Although the number of active RCs per excited cross section of PSII declined (RC/CS_O_), the efficiency of primary photochemistry of PSII was enhanced (higher F_V_/F_M_ and φP_0_). The active RCs may have benefited from the diminished dissipation of energy. It is reasonable to believe that, for the same reason, primary photochemistry has made a greater contribution to overall performance (higher φ(P_0_)/(1 − φ(P_0_))).

At 24 h, the gametophytes Fe300 showed a ChlF emission almost coincident with that of the control (Figure 2e) and JIP test (Figure 2f) returned only one significant difference, namely the lower performance index up to Q_B_ reduction (PI_ABS_) in treated samples.

Exposure of gametophytes to Fe 300 µM for 72 h affected the transient states of ChlF (Figure 2g) and JIP test (Figure 2h) highlighted that the absorbed photon flux per excited cross section of PSII was lower in treated gametophytes (lower ABS/CS_O_ ≈ F_O_). This, along with a reduced number of active RCs (lower RC/CS_O_), might have restricted the trapped exciton flux (TR_0_/CS_O_), the flux of electrons to final PSI acceptors (RE_0_/CS_O_) and energy dissipation (DI_0_/CS_M_).

After 120 h, the effects on Fe300 gametophytes were more evident: the curve of ChlF emission appeared to diverge more widely from that of the control (Figure 2i) and JIP test revealed several differences between Fe300 and control samples (Figure 2j). The treatment diminished the absorbed photon flux per excited cross section of PSII (ABS/CS_O_ ≈ F_O_ and ABS/CS_M_ ≈ F_M_), that might have lowered the trapped exciton flux (TR_0_/CS_O_ and TR_0_/CS_M_), the flux of electrons from Q_A_^−^ to Q_B_ (ET_0_/CS_M_) and the flux of dissipated energy (DI_0_/CS_M_) per excited cross section of PSII. The concomitant reduction of the number of RC per excited cross section of PSII (RC/CS_M_) may have contributed to these changes. The treatment had a positive effect on the efficiency with which an electron from reduced plastoquinone (PQH_2_) is transferred to final PSI acceptors (δR_0_).

**Figure 3:**
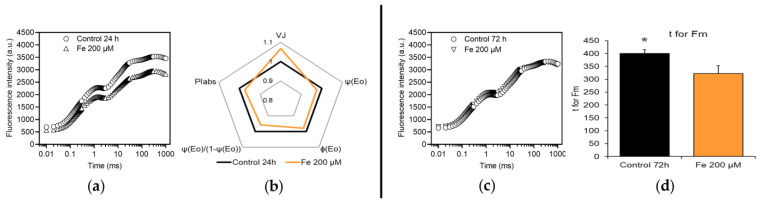
Effects of the exposure to 200 µM Fe for 24 (**a**,**b**) and 72 (**c**,**d**) h, in dark-adapted *M. polymorpha* gametophytes. Induction transients of ChlF (**a**,**c**) and spider plot (**b**) or bar chart (**d**) of parameters of JIP test (described in Table 1); values in spider plot were normalized to those of the control, which were set as one. Black line (or bar) = control; orange line (or bar) = 200 µM Fe. Only those parameters that differed significantly from the control (*, *p* < 0.05) are shown. All values are the mean of nine replications.

The treatment 200 µM Fe (Fe200) started to cause significant effects at 24 h. Fluorescence transients (Figure 3a) diverged from that of the control and JIP test (Figure 3b) showed that the efficiency (ψE_0_) and the quantum yield (φE_0_) of electron transfer from Q_A_^−^ to Q_B_ were lower in Fe200 than in control, as well as the contribution of intersystem electron transport to the global performance of photosynthesis light reactions (ψ(E_0_)/(1 − ψ(E_0_))). The slowdown of electron transport in the intersystem might be the cause of the accumulation of closed RCs, evidenced by high V_J_, and of the reduction of the performance index up to Q_B_ reduction (PI_ABS_).

The treatment Fe200 at 72 h exhibited a ChlF emission similar to the control (Figure 3c) and JIP test highlighted only one significant difference (Figure 3d), namely a lower t for F_M_ in treated gametophytes: this indicated that the maximal fluorescence F_M_ was reached faster, due to a reduced capacity to transport electrons.

### 2.3. Responses to Excess Zn

**Figure 4:**
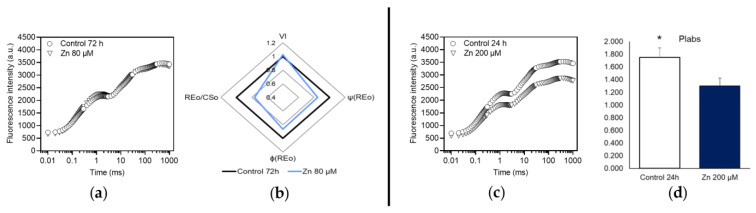
Effects of the exposure to 80 µM Zn for 72 h (**a**,**b**) and to 200 µM Zn for 24 h (**c**,**d**), in dark-adapted *M. polymorpha* gametophytes. Induction transients of ChlF (**a**,**c**) and spider plot (**b**) or bar chart (**d**) of parameters of JIP test (described in Table 1); values in spider plot were normalized to those of the control, which were set as one. Black line (or white bar) = control; pale blue line = 80 µM Zn; dark blue bar = 200 µM Zn. Only those parameters that differed significantly from the control (*, *p* < 0.05) are shown. All values are the mean of nine replications.

The overall effect of the exposure to Zn was rather mild. Gametophytes treated with 80 µM of the metal (Zn80) exhibited significant effects only at 72 h. Fluorescence transients of Zn80 samples were very similar to the control (Figure 4a), and JIP test (Figure 4b) revealed limited differences. Treated gametophytes accumulated reduced Q_B_ (higher V_I_) and the electron transport on the acceptor side of PSI was negatively affected, both per excited cross section of PSII (lower RE_0_/CS_O_) and as quantum yield (φR_0_). The latter seems to be attributable to the lower efficiency with which an exciton trapped by PSII is transferred to final PSI acceptors (ψR_0_).

Significant, yet limited, effects were observed in gametophytes treated with 200 µM Zn (Zn200) after 24 h. The pattern of ChlF emission (Figure 4c) differed between treated samples and control, but JIP test (Figure 4d) demonstrated that only PI_ABS_ was significantly different: the value was lower in treated samples, thus indicating a lower performance of energy conservation of absorbed photons up to Q_B_ reduction.

### 2.4. Overall Impact of the Treatments

**Figure 5:**
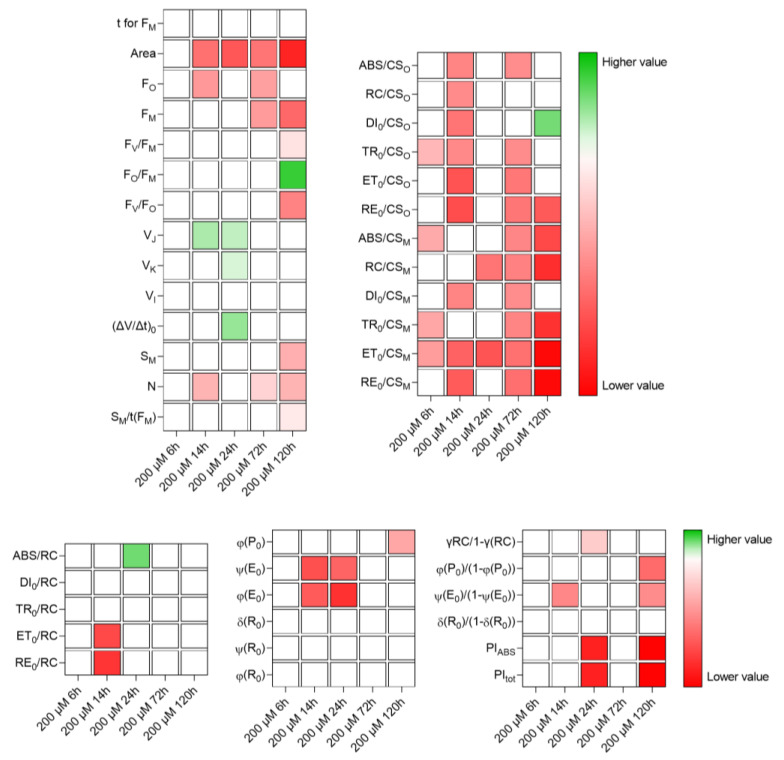
Heat map representing relative variability of the analyzed photosynthesis-related parameters, following treatment of *M. polymorpha* gametophytes with 200 µM Cu. Red is for lower values and green for the highest values. All data were first normalized to bring the value of the parameters in the range 1–100.

**Figure 6:**
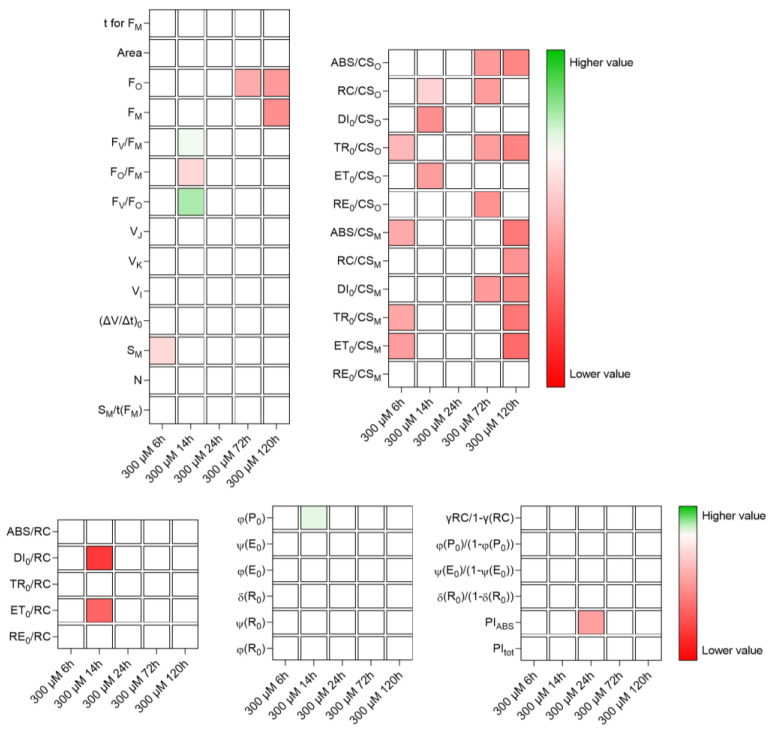
Heat map representing relative variability of the analyzed photosynthesis-related parameters, following treatment of *M. polymorpha* gametophytes with 300 µM Fe. Red is for lower values and green for the highest values. All data were first normalized to bring the value of the parameters in the range 1–100.

The treatments that produced the strongest effects on the light reactions of photosynthesis were Cu200 and Fe300. The effects of Zn were small, at both concentrations applied. The Cu80 and Fe200 treatments had minor effects, that were evident particularly after 24 h: both metals decreased the efficiency of electron transport in the intersystem and, in the case of Cu, also the electron flux in the acceptor side of PSI. The effects of Cu200 and Fe300 were summarized in Figure 5 and Figure 6, respectively.

The effects of Cu200 (Figure 5) were already evident after 6 h and appeared quite severe at 14 h. At 24 h, there seemed to be a slight recovery (as far as concerned electron flux per excited cross section of PSII), but the situation worsened again afterward (technical parameters, phenomenological energy fluxes and performance indexes). The efficiencies/quantum yields and the specific energy fluxes per active RC did not show critical values. However, after 120 h, problems began to arise also in OEC, and energy dissipation increased. At this concentration, Cu strongly reduced the flow of electrons throughout the transport chain, as well as photon absorption in the antennas.

In general, Fe300 (Figure 6) caused less severe effects than Cu200. A recovery at 24 h was apparent in Fe300 (only one parameter of JIP test was worse than the control), followed by a worsening of conditions, as indicated by the values of phenomenological energy fluxes. Modest effects were recorded on quantum efficiencies/yields and specific energy fluxes per RC. At 14 h, there were even some positive impacts on OEC, although they were transient. Part of the RCs were inactivated (as demonstrated by the decrease in RC/CS values) and the photon absorption in the antennas decreased, as well as the flow of electrons in the photosynthetic transport chain; however, the acceptor side of PSI was almost unaffected.

## 3. Discussion

Photosynthetic light reactions are particularly vulnerable to the action of heavy metals, which can cause wide-ranging effects such as thylakoid membrane disorganization, damage to OEC and impaired electron transport [8,36,37]. Using ChlF analysis, a key non-invasive technique for studying the photosynthetic apparatus in vivo, our work has shown that also in the liverwort *M. polymorpha* excessive concentrations of metal micronutrients can have significant negative impacts on photosynthesis.

The strongest effects were shown by Cu, thus substantiating the inhibition of photosynthesis by this metal that had been previously observed in some higher plants and algae [38,39]. According to the literature, Cu appears to inhibit photosynthetic electron transport, with the acceptor and donor sides of PSII indicated as the most sensitive targets [40,41,42,43]. Our study confirmed such results and added some further details. The treatment Cu200 negatively affected almost all processes of the light phase of photosynthesis, starting from the absorption of photons, up to the electron flow on the acceptor side of PSI. This metal is known to inhibit pigment accumulation, to replace Mg within the chlorophyll molecule and to hinder its integration into the photosystems. Furthermore, Cu can induce the release of proteins from inner antenna (CP47 and CP43) of PSII [44] and can inhibit the reduction of Q_A_ and Q_B_ [45]. The negative effects of Cu that we detected on electron transport in the acceptor side of PSI might be explained by the interference of the metal with ferredoxin, as previously observed in spinach [46]. In addition, when present in high concentrations and for prolonged periods of time, Cu can cause the closure also of PSI [47], beyond that of PSII. In *M. polymorpha*, the efficiencies/quantum yields and the specific energy fluxes per active RC did not attain critical values, suggesting that the components of the photosynthetic apparatus that had remained active were still functioning fairly efficiently, although they were exposed to high Cu concentrations. Despite this, at the end of the experiment the phenomenological energy fluxes displayed a marked decline of photosynthetic efficiency in *M. polymorpha*. Furthermore, OEC was negatively affected, owing probably to the known ability of Cu to inhibit the Mn cluster and/or the tyrosine TyrZ or TyrD residues [40].

The effects of Cu were far more evident at the higher concentration applied, i.e., 200 µM. The response to 80 µM Cu was much weaker and of shorter duration, and was detected only at 24 h. It altered mainly the number and activity of RCs, and the efficiency of the intersystem electron transport. In maize leaves, the same Cu concentration caused denaturation of PSII, resulting in a significant decline of electron transport [47]. *M. polymorpha* appeared to be more resistant than maize and, surprisingly, Cu had also a slight positive effect on the electron flux in the acceptor side of PSI. The reason for this is not clear and it can only be hypothesized that such positive effect might be related to the fact that Cu, being a component of plastocyanin, is actively involved in photosynthetic electron transport. Low concentrations of Cu (up to 20 µM, applied for 24 h) exhibited some beneficial effect on photosynthesis also in *Lemna minor* [48].

The role of Fe in the photosynthetic process is well documented: this metal micronutrient, owing to its function in redox reactions, is a constituent of several complexes involved in electron transport and preserves the structure and function of RCs and antenna complexes. However, excess Fe may cause toxic effects that are mediated by ROS overproduction [49]. In *M. polymorpha*, JIP test showed a stronger and earlier impact of the treatment at the highest concentration, i.e., 300 μM, while the response to 200 μM Fe was significant only after 24 h and, albeit extremely weakly, at 72 h of exposure. The treatment Fe200 at 24 h accelerated the accumulation of closed RCs and decreased the flux of electrons from Q_A_^−^ to Q_B_, while the transport in the acceptor side of PSI did not seem to be affected. The Fe300 gametophytes began to show negative effects well before Fe200. Similar to the latter, they suffered inhibition of electron transfer from Q_A_^−^ to Q_B_ and did not show negative consequences on energy fluxes and transport efficiency on the acceptor side of PSI, even after 120 h of treatment. Only at 72 h there was a decrease in the flux of electrons to final PSI acceptors per excited cross section of PSII, but it was transient and could have been an indirect consequence of the lower absorbed photon flux. Similar results were obtained in *Ipomoea batatas* L.: after exposure to 9 mM Fe for 7 days, there was a reduction in net photosynthesis, but also a positive effect of the treatment on some sections of the electron transport chain, including that on the acceptor side of PSI [50]. Other transient positive effects of Fe300 were recorded on primary photochemistry and OEC at 14 h, while lower dissipation of absorbed energy was observed at 14, 72 and 120 h. Despite that its impact was partially beneficial, Fe300 treatment had negative consequences that seemed to worsen over time, especially on photon absorption and electron flow around PSII, up to Q_B_, whereas the acceptor side of PSI was unaffected, and, after prolonged exposure, its transport efficiency was even enhanced. Overall, our data seem to confirm that Fe does not inhibit photosynthesis severely. On the contrary, at the highest concentration applied it also produced some beneficial effects, perhaps because this element is a fundamental constituent of many complexes of the photosynthetic apparatus, such as cytochromes and Fe-S clusters, as well as being an essential cofactor in the biosynthesis of these complexes and chlorophylls [51]. The impact of the treatment Fe300 was less strong than that of Cu200, but some common features were observed in the responses of gametophytes. In both cases a transient recovery occurred at 24 h, but the scenario worsened with increasing exposure times. A further common outcome of the two treatments was the mild effect on quantum efficiencies/yields and specific energy fluxes per RC: this apparently implies that the structures that kept functioning despite the treatments were still able to operate efficiently.

Zinc may have multiple negative effects on photosynthesis. Excess of this heavy metal causes reduction in photosynthetic pigments synthesis and damages the photosynthetic machinery [32]. This element replaces Mg in chlorophyll molecules [52] and may induce the release of three extrinsic polypeptides of OEC [44]. Additionally, in *Phaseolus vulgaris*, Zn has been shown to inhibit PSI and PSII and to negatively affect the synthesis of ATP [53]. Despite its potential toxicity, Zn did not exhibit all these negative effects on the gametophytes of *M. polymorpha*. The treatment Zn80 seemed to impact, only transiently (at 72 h), the electron transport beyond Q_B_, by reducing the energy flux per active PSII cross section and the efficiency of such transport, while leaving energy absorption and trapping almost unchanged. The treatment Zn200 had a negative effect only at 24 h, when it reduced the performance of energy conservation of absorbed photons up to Q_B_ reduction. Thus, it appeared that in these gametophytes mostly the electron transport chain around PSII was affected. PI_ABS_ was the only parameter of JIP test that differed from the control; therefore, it can be assumed that the effects of Zn200 on the photosynthetic apparatus were rather mild. *M. polymorpha* demonstrated a substantial resistance to Zn, but also higher plants may effectively respond to this metal: for instance, in *Beta vulgaris* treated with 50, 100, and 300 μM Zn for 10 days, only the highest concentration showed a marked inhibitory effect on photosynthesis [22]. Nevertheless, *M. polymorpha* did not seem to suffer damage to the OEC, unlike what occurred with *B. vulgaris*.

## 4. Materials and Methods

### 4.1. Plant Material

Female gametophytes of *Marchantia polymorpha* L. subsp. *ruderalis* Bischl. & Boissel.-Dub. (Marchantiales, Marchantiophyta), Cambridge-2 wild type (Cam-2, University of Cambridge, Cambridge, UK) were grown in Petri dishes, starting from the axenic cultivation of gemmae (from “gemmae cups”), in half-strength Murashige and Skoog (MS ½) medium (Duchefa Biochemie, Haarlem, The Netherlands), supplemented with 0.8% (*w*/*v*) sucrose (Duchefa Biochemie) and 0.7% (*w*/*v*) agar (Duchefa Biochemie). The MS ½ pH was adjusted to 5.7 with KOH 0.1 M. Gemmae were grown under 16:8 light/dark cycle, 19 ± 1 °C, and a photosynthetic photon flux density of 60 µmol m^−2^ s^−1^, with 60% relative humidity. After four weeks, the derived axenic gametophytes were transferred to Petri dishes for further two weeks and, subsequently, to sterile pots filled with liquid MS ½ medium (as described above, without agar) for a further two weeks. Thereafter, the eight-week-old gametophytes (about 0.8 g FW each) were individually placed under the following treatment conditions:

(1) control, with concentrations of Cu, Fe and Zn compliant to the composition of the MS medium (½), i.e., 0.05 µM CuSO_4_·5H_2_O, 50 µM FeNaEDTA and 14.95 µM ZnSO_4_·H_2_O;
(2) excess metal nutrients: Cu (as CuSO_4_), 80 µM (Cu80) and 200 µM (Cu200); Fe (as FeSO_4_), 200 µM (Fe200) and 300 µM (Fe300); Zn (as ZnSO_4_), 80 µM (Zn80) and 200 µM (Zn200).

The concentrations of the treatments were chosen by reference to the published literature. In a similar experiment [31], *M. polymorpha* gametophytes were treated with 2 mM and 200 µM of either Cu or Zn: at the highest concentration, the two metals caused extensive mortality; therefore, we chose 200 µM for our treatments. The maximum level of Fe was chosen based on the results of [54] on *M. polymorpha*. The lowest concentrations were selected with the aim of characterizing the response of the plant to levels of Cu, Fe and Zn intermediate between those of the growth medium (i.e., the control) and the maximum ones that had been established previously. Gametophytes were treated for 6, 14, 24, 72 and 120 h. Four biological replicates for each sampling time were prepared for all treatments.

### 4.2. Chlorophyll a Fluorescence Transient Kinetic and OJIP Parameters

The overall functional efficiency of plants was investigated by the analysis of the biophysics of the photosynthetic light reactions. Such evaluation was performed by the measurement of PSII fluorescence. This was recorded, at the aforementioned times, by a chlorophyll fluorometer (Handy PEA, Hansatech Instruments Ltd., Pentney, King’s Lynn, UK). Gametophytes were harvested, dried on blotting paper and part (two or three spots) of their surface was darkened with specific clips for 30 min. Nine measurements were then taken for each treatment: two from each of three gametophytes (replications), and three from the fourth replication. The darkened spots were exposed for 1 s to 3500 μmol photons m^−2^ s^−1^ (650 nm peak wavelength) and ChlF was recorded. Data were processed by PEA plus software (Hansatech Instruments Ltd., King’s Lynn, UK), which performed the analysis of the fast fluorescence kinetics, i.e., JIP test [55]. The recordings from each gametophyte were averaged to yield a single value, which was then treated as an independent replication. The JIP test parameters were calculated from ChlF values recorded at 50 μs, 100 μs, and 300 μs, along with F_O_, F_J_, F_I_, and F_M_ [32]. The parameters are listed in Table 1.

**Table 1:** Definition of the measured ChlF values and of the calculated parameters of JIP test for the analysis of the fast transient states of ChlF. O = origin (minimum fluorescence, F_O_), J and I are intermediate states at 2 and 30 ms (F_J_ and F_I_), respectively, and P = peak (maximum fluorescence, F_P_ or F_M_). PSI = photosystem I; PSII = photosystem II; RC = total number of reaction centers within the gametophyte spot measured; CS = excited PSII cross section; Q_A_ = primary quinone acceptor of PSII; Q_B_ = secondary quinone acceptor of PSII; OEC = oxygen evolving complex of PSII [56].

Technical Parameters of Fluorescence	Definition
F_O_	Fluorescence value (minimum) after the onset of illumination
F_M_	Fluorescence value at the peak of OJIP curve; maximum value under saturating illumination
F_V_ = F_M_ − F_O_	Maximum variable fluorescence
F_O_/F_M_	Maximum quantum yield at t = 0 of energy dissipation
F_V_/F_O_	Maximum efficiency of the reaction of photolysis of water; it is a proxy of the integrity of OEC
F_V_/F_M_	Maximum quantum yield of primary PSII photochemistry
V_t_ = (F_t_ − F_O_)/F_V_	Relative variable fluorescence
V_J_	Relative variable fluorescence at J state (2 ms), which is a proxy of the number of closed RCs
V_K_	Relative variable fluorescence at K state (300 µs); it rises when OEC breaks down
V_I_	Relative variable fluorescence at I state (30 ms), which is a proxy of the number of reduced Q_B_
t for F_M_	Time (in ms) to reach maximal fluorescence F_M_
N	Turnover number: number of Q_A_ reduction events between t = 0 and t(F_M_)
M_0_ = (ΔV/Δt)_0_ ≈ 4(F_0.3ms_ − F_0.05ms_)/F_V_	Initial slope (ms⁻^1^) of fluorescence rise in O–J; it is a proxy of the rate of accumulation of closed RCs
Area	Area between the OJIP curve and the line F = F_M_, which is a proxy of the number of Q_A_ acceptors
S_M_ = Area/F_V_	Normalized area between the OJIP curve and the line F = F_M_, which is a proxy of the number of electron carriers per electron transport chain
S_M_/t(F_M_)	Expresses the average fraction of open RCs in the time span from 0 to t(F_M_), i.e., during the time needed to complete their closure
**Energy fluxes**	**Definition**
ABS	The photon flux absorbed by the antenna of PSII units
TR	The part of ABS trapped by the active PSII units that leads to Q_A_ reduction
DI	The part of ABS dissipated in PSII antenna in processes other than trapping
ET	The energy flux associated with the electron transport from Q_A_^−^ to the intersystem electron acceptors
RE	The energy flux associated with the electron transport to the final electron acceptors of PSI
**Efficiencies and quantum yields**	**Definition**
ET_0_/TR_0_ = ψE_0_ = 1 − V_J_	Efficiency with which a PSII trapped electron is transferred from Q_A_^−^ to PQ
RE_0_/TR_0_ = ψR_0_ = 1 − V_I_	Efficiency with which a PSII trapped electron is transferred to final PSI acceptors
RE_0_/ET_0_ = δR_0_ = ψR_0_/ψE_0_	Efficiency with which an electron from PQH_2_ is transferred to final PSI acceptors
TR_0_/ABS = φP_0_ = F_V_/F_M_	Maximum quantum yield of primary PSII photochemistry
ET_0_/ABS = φE_0_ = φP_0_ × ψE_0_	Quantum yield of electron transport from Q_A_^−^ to PQ
RE_0_/ABS = φR_0_ = φP_0_ × ψR_0_	Quantum yield of electron transport to final PSI acceptors
γRC = 1/[(ABS/RC) + 1] = RC/(ABS + RC)	Probability that a PSII chlorophyll molecule functions as RC
DI_0_/ABS = 1 − TR_0_/ABS	Quantum yield of energy dissipation in PSII
**Specific energy fluxes** **(per active PSII)**	**Definition**
ABS/RC = (M_0_/V_J_)/φP_0_	Apparent antenna size of an active PSII
TR_0_/RC = M_0_/V_J_	Maximum trapped exciton flux per active PSII
ET_0_/RC = (M_0_/V_J_) × ψE_0_	The flux of electrons transferred from Q_A_^−^ to PQ per active PSII
RE_0_/RC = (M_0_/V_J_) × ψR_0_	The flux of electrons transferred to final PSI acceptors per active PSII
DI_0_/RC = ABS/RC − TR_0_/RC	The flux of energy dissipated in processes other than trapping per active PSII
**Phenomenological energy fluxes** **(per CS)**	**Definition**
ABS/CS_O_ ≈ F_O_ and ABS/CS_M_ ≈ F_M_	Absorbed photon flux per excited cross section of PSII
RC/CS = (RC/ABS) × (ABS/CS)	Number of active RCs of PSII per excited cross section of PSII
TR_0_/CS = (TR_0_/ABS) × (ABS/CS)	Maximum trapped exciton flux per excited cross section of PSII
ET_0_/CS = (ET_0_/ABS) × (ABS/CS)	The flux of electrons from Q_A_^−^ to PQ per excited cross section of PSII
RE_0_/CS = (RE_0_/ABS) × (ABS/CS)	The flux of electrons to final PSI acceptors per excited cross section of PSII
DI_0_/CS = (ABS/CS) − (TR_0_/CS)	Flux of dissipated energy per excited cross section of PSII
**Performance indexes**	**Definition**
φ(P_0_)/(1 − φ(P_0_))	Partial performance of primary photochemistry reactions, i.e., their contribution to the global performance of photosynthesis light reactions
γRC/(1 − γRC)	Number of active RCs per antenna chlorophyll of PSII
ψ(E_0_)/(1 − ψ(E_0_))	Partial performance of intersystem electron transport, i.e., its contribution to the global performance of photosynthesis light reactions
δR_0_/(1 − δR_0_)	Partial performance of electron transport from Q_B_ to the final acceptors of PSI, i.e., its contribution to the global performance of photosynthesis light reactions
PI_ABS_	Performance index of energy conservation of absorbed photons up to Q_B_ reduction
PI_tot_	Performance index of energy conservation of absorbed photons up to reduction of the final acceptors of PSI

### 4.3. Statistical Analyses

The data were first checked for normality of distribution (by Shapiro–Wilk test) and homogeneity of variances (by Levene test). The values of ChlF and the parameters of JIP test were compared between each treatment and the respective control, at each time, by Student’s t test. The level of significance was *p* < 0.05 (*). Statistical analyses were performed by Past 4.06b [57] and graphs were drawn by Microsoft Excel 2016 and GraphPad Prism 9.

## 5. Conclusions

Our data demonstrate that high concentrations of metal micronutrients such as Cu, Fe, and Zn impact the photosynthetic machinery of the liverwort *M. polymorpha*. The mechanisms of action and the extent of negative effects depend on the element and its concentration. Copper, especially at the highest concentration, disrupted the entire electron transport chain, whereas Fe had negative effects mainly around PSII, that were less severe than those of Cu. The effects of Zn were even weaker and of shorter duration.

Given the scarcity of data available on the response of photosynthesis to metal micronutrients in experimental systems other than higher plants, and given the efficiency with which organisms such as *M. polymorpha* absorb chemical elements from the environment, the present study may represent a starting point for further investigations on the effects of Cu, Fe, and Zn and on the mechanisms underlying heavy metal detoxification and tolerance in bryophytes. This represents basic knowledge to elucidate the evolution of the biochemical and molecular processes that confer to land plants resistance to heavy metals and to develop novel and effective biomonitoring techniques for the protection of the environment.

## Data Availability

Not applicable.
